# Effects of Finishing on Surface Roughness of Four Different Glass-Ionomer Cements and One Alkasite: In Vitro Investigation over Time Using Aging Simulation

**DOI:** 10.3390/jfb15110325

**Published:** 2024-10-31

**Authors:** Alexander Behlau, Isabelle Behlau, Michael Payer, Gerd Leitinger, Katharina Hanscho, Lumnije Kqiku, Karl Glockner

**Affiliations:** 1 Division of Restorative Dentistry, Periodontology and Prosthodontics, Dental Medicine and Oral Health, Medical University of Graz, 8010 Graz, Austria; katharina.hanscho@medunigraz.at (K.H.); lumnije.kqiku@medunigraz.at (L.K.); karl.glockner@medunigraz.at (K.G.); 2 Department of Psychology, Bundeswehr University Munich, 85577 Neubiberg, Germany; isabelle.behlau@unibw.de; 3 Division of Oral Surgery and Orthodontics, Dental Medicine and Oral Health, Medical University of Graz, 8010 Graz, Austria; michael.payer@medunigraz.at; 4 Division of Cell Biology, Histology and Embryology, Research Unit Electron Microscopic Techniques, Gottfried Schatz Research Center, Medical University of Graz, 8010 Graz, Austria; gerd.leitinger@medunigraz.at

**Keywords:** amalgam-replacement materials, optical surface measurement, European amalgam ban, non-contact profilometer, surface roughness

## Abstract

In 2017, Europe implemented a ban on amalgam restorations for children aged <15 years and for pregnant/breastfeeding women, highlighting the need for alternative filling materials exhibiting less surface roughness and enhanced longevity. This in vitro study aimed to examine the surface roughness variations of five amalgam-replacement materials across three time points and using six finishing methods: (1) no finishing (control), (2) Arkansas burs, (3) diamond burs, (4) tungsten carbide burs, (5) SofLex discs in descending grit size, and (6) coarse SofLex discs combined with silicone polishing. We prepared 960 samples. Each material group, i.e., Cention Forte (CNF), DeltaFil (DLF), Ketac Universal (KTU), IonoStar Molar (ISM), and Equia Forte HT (EQF), comprised 60 samples (n = 10 per finishing method) created using standardized 3D-printed metal molds. Surface roughness (Sa) was measured immediately after finishing, after 30 days of storage in distilled water, and after thermocycling (5000 cycles) using a non-contact profilometer. The results indicate that conventional and hybrid glass-ionomer cements have smoother surfaces than high-viscosity GICs. The DLF and CNF groups exhibited stable outcomes. These findings underscore the importance of selecting appropriate finishing methods based on the restorative material to minimize surface roughness.

## 1. Introduction

Amalgam has historically been a commonly used restorative material in the field of conservative dentistry. However, in 2017, following the completion of the Minamata convention in 2013, the European Union banned the use of amalgam dental fillings in children under 15 years of age and pregnant/breastfeeding women due to the increased risk of mercury release and fear of amalgam [1]. This highlighted the need for an evaluation of the properties (e.g., surface roughness) of amalgam-replacement materials such as bulk-fill composite resins and glass-ionomer cements (GICs) to gain insights into their longevity. Alkasites are a newly developed composite restorative material exhibiting moderate viscosity and high mechanical resilience [2]. The monomers of these materials are primarily made up of a combination of four dimethacrylates, with urethane dimethacrylate (UDMA) being the main component. Alkasites have a 1-year survival rate of 92.5–100% [3,4].

Furthermore, GICs are often recommended as a replacement for amalgam. Research has shown that GICs are more suitable for single-surface fillings compared to amalgam [5]. Highly viscous glass-ionomer cements, the composition of glass hybrids, and the incorporation of elastomeric micelles are recent advancements in GIC developments [6,7]. They have a 2-year survival rate of 100% and a 6-year survival rate of 92.3% [8,9]. The surface roughness of restorations plays an essential role in their longevity, with evidence suggesting that lower (i.e., an arithmetic average (Ra) of <0.2 μm)) and higher surface-roughness values were associated with decreased and increased bacterial adhesion, respectively [10]. The latter has also been associated with an increased risk of developing dental caries or periodontal diseases [11]. A previous clinical trial conducted over a period of 2 years found that approximately 14% of teeth exhibiting molar incisor hypomineralization and those restored using high-viscosity GICs developed secondary caries [12].

Although manufacturers typically claim that GIC restorations do not require polishing, finishing of the restoration contour and occlusion should be considered essential. Existing evidence on GICs considers a limited number of restorative materials and finishing methods only, with studies often reporting contradictory findings when examining tungsten carbide burs with a varying number of blades, diamond burs, Arkansas burs, and SofLex discs [13,14].

Evidence also suggests that the surface roughness of restorations increases over time [15]. Thermocycling, which aims to simulate the thermal stresses observed within the oral cavity, has been shown to increase the surface roughness of two composites, bearing a direct association with the number of thermocycles being observed [16]. Although GICs have historically been used as a temporary restorative material, the WHO recently recommended its use as an alternative for amalgam [17]. However, no studies, to date, have examined the effects of thermocycling and varying thermal stresses (i.e., different thermal expansion coefficients) on the surface roughness of GIC restorations [18]. Therefore, the current study aimed to compare the surface roughness of five amalgam-replacement materials over time and by the finishing methods used. Thermocycling was used to simulate restoration aging in vitro to allow for the examination of changes in surface roughness over time. The null hypothesis being tested was that the bulk-fill materials would not exhibit any differences in surface roughness over time and by the finishing methods used.

## 2. Materials and Methods

**Figure 1:**
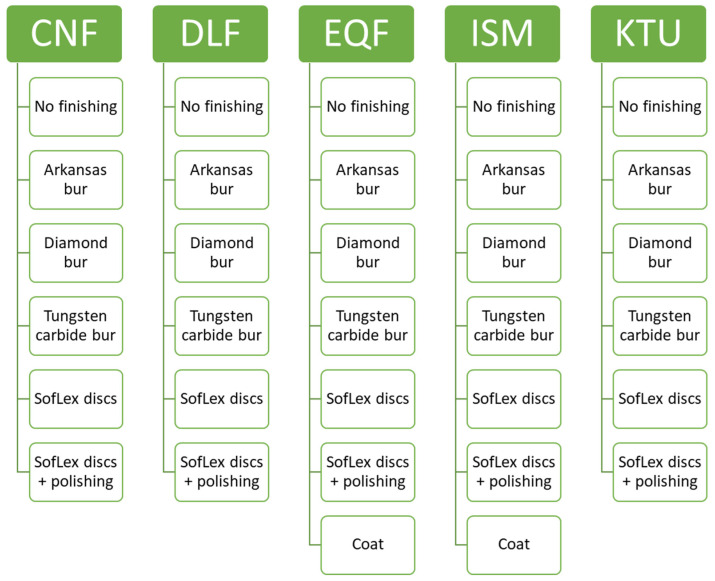
Grouping of samples.

The current study examined five self-curing amalgam-replacement materials, including an alkasite (i.e., Cention Forte (CNF), Ivoclar Vivadent AG, Schaan, Liechtenstein); a conventional GIC (i.e., IonoStar Molar (ISM), Voco, Cuxhaven, Germany); a conventional GIC with micelles technology (i.e., DeltaFil (DLF), DMG, Hamburg, Germany); a high-viscosity GIC (i.e., Ketac Universal (KTU), 3M ESPE, St Paul, MN, USA); and a glass hybrid (i.e., Equia Forte HT (EQF) GC Dental Products Corp., Tokyo, Japan) (Figure 1). All materials are bulk-fill materials without height limitations. Further details of the material properties are provided in Table 1.

Each of the five material groups contained 60 samples (length × height × width: 3 × 3 × 3 mm), and comparisons were carried out at three time points (i.e., immediately after finishing, after 30 days of storage in distilled water, and after thermocycling) and by six finishing methods (n = 10 each; Table 2). In addition, a coat without finishing was applied immediately after setting, as recommended by the manufacturer’s instructions for EQF and ISM (10 samples each). The coating was polymerized for 20 s in soft-start mode with a light intensity of 2000–2200 mW/cm using Bluephase 20i (Ivoclar Vivadent AG, Schaan, Liechtenstein). Therefore, a total of 960 (320 samples × 3 time points) comparisons were performed.

**Figure 2:**
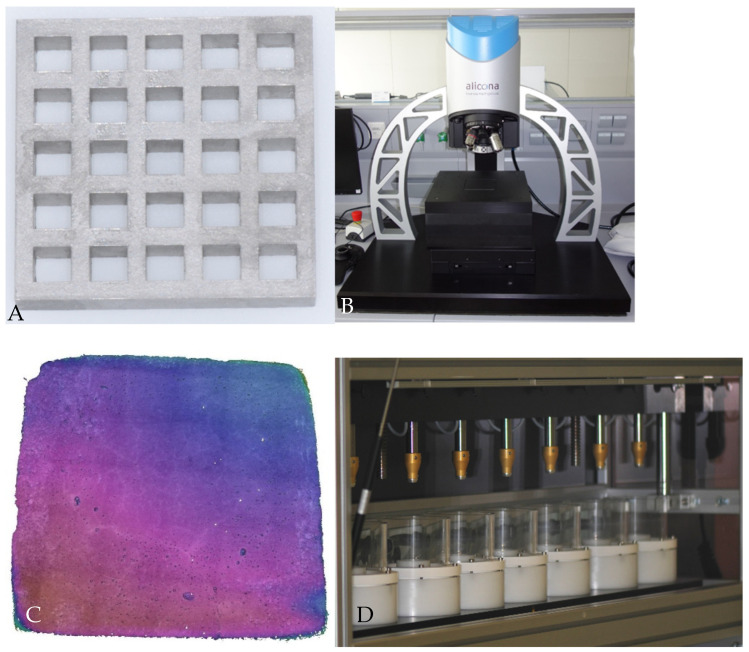
Overview of measurements carried out. (**A**) Three-dimensional-printed metal mold; (**B**) optical surface roughness measurement device (Infinite Focus G5, Alicona); (**C**) representative picture of field measurement for analyzing surface roughness (Sa, in μm); (**D**) thermocycler used to simulate aging in the restorations.

The samples were created by first placing the material in quadratic metal molds created using three-dimensional (3D) printing, and any excess material was removed from the metal mold using a razor blade (Figure 2A). To provide standardized samples, glass slides were used on the bottom and top of the metal mold to ensure that the samples were free of air bubbles. The materials were then self-cured for 5 min, as per the manufacturer’s instructions; removed from the mold; and randomly divided into six subgroups based on the type of finishing method used, as follows: (1) no finishing (control group); (2) cylindrical Arkansas bur (Komet Dental, Lemgo, Germany); (3) cylindrical diamond bur (Intensiv, Montagnola, Swiss); (4) cylindrical tungsten carbide bur (Komet Dental, Lemgo, Germany); (5) SofLex discs (3M ESPE, St Paul, MN, USA) used in descending grit size; and (6) coarse SofLex discs combined with silicone polishing (Figure 1). Finishing was performed immediately after sample preparation. The spindle rotation speed was determined based on the manufacturer’s instructions, and water cooling was carried out at a minimum rate of 50mL/min. All samples were finished once from the left to the right direction by a single operator to ensure equal distribution of pressure, and a randomized protocol was used for instrumentation to minimize training effects. To ensure that no training effects from finishing influenced the results, finishing was performed randomly within each material group. For example, for CNF, the order of finishing groups was (3), (5), (1), (2), (6), and (4), while for DLF, the order was (4), (6), (5), (2), (3), and (1).

### 2.1. Surface Roughness Measurement

Surface roughness was evaluated using a 3D high-resolution optical surface measurement system (Infinite Focus G5, Alicona Imaging GmbH, Grambach, Austria; magnification: 50×; maximum resolution: 20 nm; Figure 2B) that uses light reflections to vertically scan a surface, calculate the spatial coordinates, and produce a digital copy of the sample [20,21]. Pictures of field measurements were used to examine the entire surface of the sample, which means they were 3 mm × 3 mm in size (Figure 2C), and to determine the mean surface roughness (Sa) in μm. Changes in the surface roughness over time were evaluated using the same process after storing all samples in distilled water for 30 days at a temperature of 37 °C and by exposing them to thermocycling [13,14,22].

### 2.2. Thermocycling

After 30 days, all samples underwent thermal cycling (Thermocycling TC-4, SD Mechatronik GmbH, Feldkirchen-W., Germany; Figure 2D). Based on previous research, samples were alternately immersed in cold (5 °C) and warm (55 °C) water at intervals of 2 min for a total of 5000 cycles [23].

### 2.3. Statistical Analysis

All statistical analyses were performed using the statistical software SPSS 27 (Chicago, IL, USA). After confirming normality of the data distribution using the Shapiro–Wilk test, a univariate analysis of variance was used to compare the surface roughness by bulk-fill material and the finishing method used. A post hoc analysis using the Bonferroni correction was carried out to confirm the association between groups (i.e., bulk-fill materials and finishing systems, respectively). Variables that did not exhibit a normal distribution were examined using the Kruskal–Wallis test, followed by a post hoc analysis using the Mann–Whitney U test. A *p* value of <0.05 was considered statistically significant.

## 3. Results

**Figure 3:**
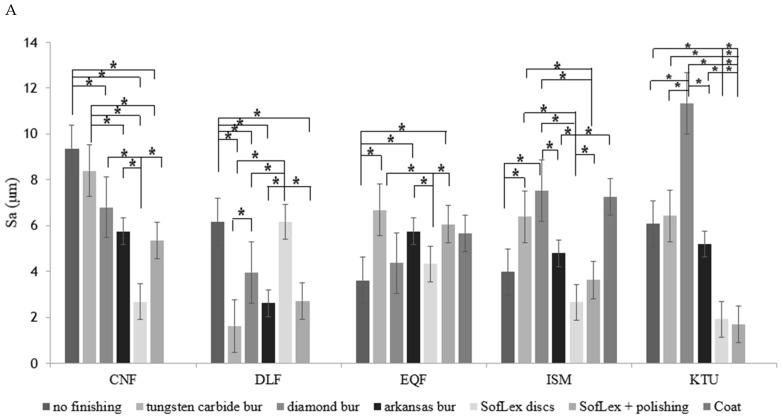

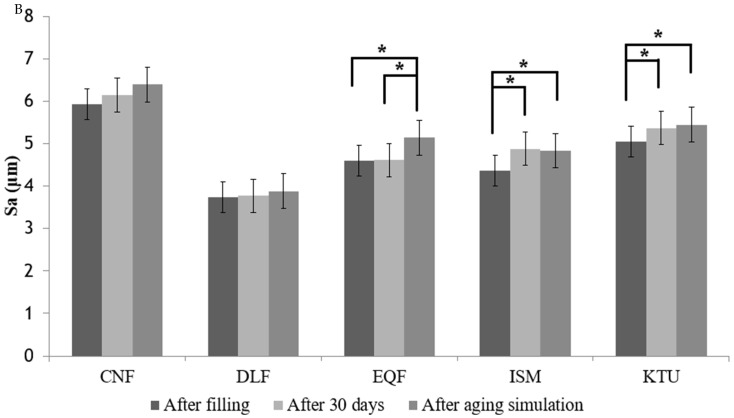
Surface roughness analysis. (**A**) Mean difference in surface roughness of five restorative materials by the finishing methods used after thermocycling. (**B**) Mean differences in surface roughness of restorative materials over time. Asterisks indicate significant differences.

The first analysis focused on differences in surface roughness by the finishing method used at time point three to analyze long-term effects. All five bulk-fill materials exhibited significant differences in surface roughness by the finishing method used (*p* < 0.001). The post hoc analysis of the CNF group showed that the subgroups of SofLex and SofLex with additional polishing exhibited smoother surfaces after thermocycling than the subgroups of no finishing and tungsten carbide burs. Moreover, the subgroups of diamond burs and Arkansas burs exhibited smoother surfaces than the subgroups of no finishing and tungsten carbide burs, respectively. Finally, the SofLex subgroup exhibited smoother surfaces than the subgroups of diamond burs, Arkansas burs, and SofLex with additional polishing (Table 3 and Table 4 and Figure 3A, Supplementary Materials).

In the DLF group, the subgroups of tungsten carbide burs, diamond burs, Arkansas burs, and SofLex with additional polishing exhibited smoother surfaces after thermocycling than the no-finishing and SofLex subgroups. Moreover, the tungsten carbide bur subgroup exhibited smoother surfaces than the diamond bur subgroup (Table 3 and Table 4 and Figure 3A).

In the EQF group, the no-finishing subgroup exhibited smoother surfaces after thermocycling than the subgroups of tungsten carbide burs, Arkansas burs, and SofLex with additional polishing. Moreover, the SofLex subgroup exhibited smoother surfaces than the subgroups of tungsten carbide burs, Arkansas burs, and SofLex with additional polishing (Table 3 and Table 4 and Figure 3A).

In the ISM group, the SofLex subgroup exhibited smoother surfaces after thermocycling than the subgroups of tungsten carbide burs, diamond burs, Arkansas burs, and SofLex with additional polishing or coating, while the subgroup of SofLex with additional polishing exhibited smoother surfaces than the tungsten carbide bur or diamond bur subgroups (Table 3 and Table 4 and Figure 3A). Finally, the no-finishing subgroup exhibited smoother surfaces than the tungsten carbide bur and diamond bur subgroups, while the Arkansas bur subgroup exhibited smoother surfaces than the diamond bur subgroup.

In the KTU group, the subgroups of SofLex and SofLex with additional polishing exhibited smoother surfaces after thermocycling than the subgroups of no finishing, tungsten carbide burs, diamond burs, and Arkansas burs. Moreover, the subgroups of no finishing, tungsten carbide burs, and Arkansas burs exhibited smoother surfaces than the diamond bur subgroup (Table 3 and Table 4 and Figure 3A).

A comparison of the surface roughness over time (i.e., after finishing; after 30 days of storage in distilled water; and after thermocycling), irrespective of the finishing method used, also showed significant differences in surface roughness (*p* < 0.001), with the EQF (*p* < 0.05) and DLF (*p* < 0.001) groups exhibiting smoother surfaces than the CNF group immediately after finishing and after 30 days of storage in distilled water. Moreover, the DLF group exhibited smoother surfaces than the KTU group after 30 days of storage in distilled water (*p* < 0.05), and this difference remained statistically significant even after the samples had undergone thermocycling (*p* < 0.05). Similar findings were observed upon comparison of the DLF and CNF groups after thermocycling (*p* < 0.001; Figure 3B). No other statistically significant differences were observed between the bulk-fill materials examined.

An evaluation of the differences over time, irrespective of the finishing method used, showed stable surface roughness in the CNF and DLF groups (*p* > 0.05). In contrast, the EQF group exhibited smoother surfaces immediately after finishing and after 30 days of storage in distilled water when compared to after thermocycling (*p* < 0.001; Figure 3B). The ISM and KTU groups exhibited smoother surfaces immediately after finishing compared to after 30 days of storage in distilled water and thermocycling (*p* < 0.05; Figure 3B).

## 4. Discussion

Dental fillings often require replacements due to bulk fractures, the loss of restorative materials, and microleakages caused by secondary caries [22,24]. Previous studies in this field have primarily focused on the antimicrobial effects of fluoride release by restorative materials (including those examined in the current study) on the risk of secondary caries and microleakage, with evidence showing that lower doses of fluoride release, as observed in compomers, have no effects on bacterial growth [25]. However, several types of restorations (e.g., crowns, composites, and amalgam fillings) do not exhibit any fluoride release, suggesting that their surface roughness may play a significant role in caries prevention and longevity. Evidence suggests that surface-roughness values of >0.2 μm (Ra) are associated with more plaque accumulation, while smoother surfaces are associated with lower shear forces and, consequently, bacterial adhesion [10,26,27].

The current study examined variations in the surface roughness of five bulk-fill materials over time and by the finishing methods used. The findings show that the surface roughness of GICs increased over time, with the exception of the DLF and CNF groups, which remained stable. These findings are in agreement with evidence from previous studies [15]. Manufacturers have made significant improvements in amalgam-replacement materials, with CNFs and DLFs exhibiting improved surface roughness due to the presence of dimethacrylates and PEG-PU micelles in their compositions, respectively [2,28]. Particle size can also have beneficial effects on surface roughness over time, with a previous study demonstrating that composites with smaller particles exhibit smoother surfaces [29]. The filler sizes of composites have often been investigated, leading to a classification of nano-, micro-hybrid, and microcomposites [30]. Such a classification does not exist for GICs. However, previous studies have shown that the particle size of Cention Forte ranges from 0.1 to 7 µm, while the particle size of Ketac Universal is 7.2 µm [31,32]. For Equia Forte HT, prior research indicated that this material is composed of ultrafine particles [33]. Additionally, small particles are associated with lower surface-roughness values in composites, which is also assumed for GICs [15,34]. Smaller particles tend to have higher strength values but lower wear-resistance values [35,36]. Furthermore, GICs with nano-fillers are more prone to damage by thermocycling than conventional GICs [37]. Thus, particle size is an influential factor for GICs. The present results indicate that smaller particles are easier to finish for GICs, resulting in smoother surfaces. This finding is consistent with those of prior studies that show that GICs with added nano-fillers have improved mechanical properties like flexural strength [37].

The differences in surface roughness observed in the current study could potentially be attributed to the non-uniformity of finishing materials containing inorganic fillers [38]. Glass hybrids (e.g., EQF) containing smaller particles exhibited lower surface-roughness values than those of alkasites such as CNF, while high-viscosity GICs (e.g., KTU) containing larger particles exhibited greater surface roughness than conventional GICs (e.g., DLF). Future studies should aim to examine the effects of the filler particle size on the mechanical properties (e.g., surface roughness) of restorative materials.

Moreover, future research should focus on the distribution of particles, for example, in high-viscosity GICs, which include two types of fillers: small and large. Thus, focus should be on the average particle size, because previous studies have already shown that Fuji IX (GC Dental Products Corp., Tokyo, Japan) is an example of a GIC with two roughness peeks. Hence, if two types of fillers are expected with two different means, one single average value cannot represent reality [39].

The DLF group exhibited greater stability in surface smoothness over time, particularly in comparison to the CNF and KTU groups, and this was in contradiction with a previous study that found that composites and GICs exhibit greater surface roughness after a period of 3 months [40]. However, GICs with acrylic–maleic acid undergo a maturation process up to 3 weeks. Polyacrylic acid produces fewer crosslinked chains, and they are thus less brittle than acrylic/maleic acid [41]. This could explain why KTU, the only material with acrylic/maleic acid tested, was smooth only at time point 1. Additionally, the PEG-PU micelles in DLF, as well as the hybrid glass particles in EQF, ensure that these materials smoothen over time, in contrast to ISM, which lacks such additional components, which may be the reason why ISM initially appears smooth but may develop greater porosity over time.

Previous studies reported no significant differences in the surface roughness of GICs finished using tungsten carbide burs and diamond burs, as well those finished using tungsten carbide burs with a varying number of blades. However, the Arkansas bur has been associated with greater surface roughness [13,14]. Another study showed that SofLex discs produce smoother surfaces than diamond burs and tungsten carbide burs, although the surface roughness of the samples increase over time (i.e., immediately after preparation, after 24 h, and after 7 days) [15].

The findings of the current study suggest that the optimal finishing method varies with the restorative material used, with smoother surfaces being observed when using Arkansas burs and SofLex discs in the CNF and ISM groups; tungsten carbide burs, Arkansas burs, and SofLex discs in the KTU group; tungsten carbide burs in the DLF group; and SofLex discs in the EQF group. These results are consistent with those of previous studies that showed that diamond burs are related to the greater surface roughness of composites. This has been attributed to the grain size of these burs in the past [42,43,44]. In contrast, SofLex discs were frequently associated with smooth surfaces of composites in previous studies [44,45,46]. Thus, we can replicate these results for GICs. Additionally, our results suggest that tungsten carbide bur, which is harder than the other finishing methods investigated, may be necessary to produce smooth surfaces of materials with harder particles, such as KTU with larger particles or DLF with added PEG-PU micelles. This result is consistent with those of previous studies which demonstrated that the finishing methods of composites must be harder than the filler of these material to be effective [47]. GICs are only smooth and flowable in the initial setting reaction. After the initial setting reaction, aluminum polyacrylate is formed during the maturation process to ionically crosslinked chains, which lasts for days, up to a month. The material is only solid when these chains are built [41]. The results of the present study suggest that Arkansas bur is especially suitable for filling materials with a longer initial setting reaction like for CNF, ISM, and KTU.

Furthermore, the coatings did not appear to have any beneficial effects on surface roughness over time or after thermocycling, although this finding is in contrast to a previous study that showed that uncoated restorations exhibit greater surface roughness than coated restorations [48]. The EQF group, in particular, exhibited stable surface roughness immediately after finishing and after 30 days of storage in distilled water, although this was seen to increase after thermocycling, potentially due to the loss of the surface coating. The current study compared changes in restorative materials after an aging simulation, and this is in contrast with previous studies that compared brushed and unbrushed fillings [48]. Although unpolished surfaces are expected to be smoother than finished surfaces, previous studies have shown that unpolished surfaces are actually less smooth [49].

A majority of relevant studies, to date, have estimated mean surface roughness by using a contact profilometer with a diamond tip to scratch scan lines in five predefined areas on the surface of the sample [13,14,15]. However, this method has the obvious limitation of an increased risk of surface damage, which can often remain undetected [50]. Furthermore, an evaluation of lines facilitates measurements along a single profile only, making the detection of very steep or negligible irregularities challenging [51]. These limitations make the examination of changes in the same surface over time difficult, and measurements of the predefined areas only can lead to unreliable findings. In addition, stylus profilometry has limitations in the spatial dimension by analyzing microgrooves, so the measured surface could be smoother [21,52]. Previous research has shown a heightened range for amplitude measurements with optical scanning [53]. These limitations have been addressed to a certain extent by non-contact profilometry, which is becoming increasingly popular for the evaluation of surface roughness [52]. Another recent improvement in this domain is the measurement of the entire surface (Sa) instead of surface lines (Ra) only [54]. In accordance with existing evidence, the current study shows that the mean Sa values tend to be higher than the mean Ra values [55]. The Sa values in this study vary between two and 11 µm. In contrast, previous research showed Ra values between one and five µm for GICs [56].

One of the greatest strengths of this study is its extensive comparison of different amalgam-replacement materials and finishing methods over time, which is lacking in previous research. This study aimed to minimize limitations by using standardized methods for all investigated samples. Clinically, the results imply the importance of carefully selecting restorative materials and appropriate finishing methods, as these choices are crucial for maintaining long-term smooth surfaces. However, replicating the complex oral environment presents a limitation. The results of this study could be influenced by masticatory forces, shifts in the oral microbiome, or fluctuations in pH. Furthermore, this study focused on flat surfaces, while real tooth surfaces have cusps and valleys that impede uniform finishing. Therefore, the present findings should be substantiated in vivo in future studies.

## 5. Conclusions

The differences in surface roughness observed in the current study highlight the importance of selecting finishing methods based on the restorative material being used. Arkansas burs, tungsten carbide burs, and SofLex discs yielded superior results in terms of surface roughness when used with restorative materials containing smaller (e.g., conventional or hybrid GICs) rather than larger (e.g., high-viscosity GIC) particles. The addition of PEG-PU micelles to GICs enhanced the long-term smoothness of the restoration surfaces, while the use of surface coatings did not have any beneficial effects on the latter. Therefore, clinicians should select the finishing method based on the restorative material being used in order to minimize surface roughness. Moreover, patients will benefit from improved long-term stability of restorations and a decreased risk of secondary caries when inappropriate finishing methods are avoided.

## Figures and Tables

**Table 1:** Bulk-fill materials examined in this study.

Code	Material	Type	Pretreatment Y/N	Curing Method	Manufacturer	Composition	Lot Number
CNF	Cention Forte	Self-curing * alkasite	Yes	Dual	Ivoclar Vivadent AG, Schaan, Liechtenstein	Powder: barium aluminum silicate glass, ytterbium trifluoride, isofiller, calcium barium aluminum fluorosilicate glass, and calcium fluoro silicate glass. Liquid: urethane dimethacrylate, tricyclodecandimethanol dimethacrylate, tetramethyl-xylylene diurethane dimethacrylate, polyethylene glycol 400 dimethacrylate, ivocerin, and hydroxyperoxide.	Z00HCG
DLF	DeltaFil	c-GIC	Yes	Self	DMG, Hamburg, Germany	Powder: fluoroaluminosilicate glass and polyacrylic acid. Liquid: polyacrylic acid, tartaric acid, PEG-PU micelles, and water.	242394
EQF	Equia Forte HT	Hybrid-GIC	No	Self	GC, Tokyo, Japan	Powder: fluoroalumino silicate glass, hybrid glass particles, and polyacrylic acid powder. Liquid: polyacrylic acid, polybasic carboxylic acid, and distilled water.	200121A
ISM	IonoStar Molar	c-GIC	No	Self	Voco, Cuxhaven, Germany	Powder: fluoro-alumino-silicate glass, polyacrylic acid powder, and pigment. Liquid: polyacrylic acid, tartaric acid, and distilled water.	2021115
KTU	Ketac Universal	Hv-GIC	No	Self	3M ESPE, St Paul, MN, USA	Powder: oxide glass. Liquid: copolymer of acrylic acid, acrylic–maleic acid, tartaric acid, benzoic acid, and water.	7797952

c: conventional; GIC: glass ionomer cement; Hv: high viscosity; * optional light-curing mode. PEG-PU is a polymer of polyurethane-polyethylene glycol. In aqueous solutions, it can self-assemble into micelles [19].

**Table 2:** Finishing systems examined in this study.

Type	Manufacturer	Grit Size	Rotation Speed
No finishing (control group)	-	-	-
Arkansas bur	Komet Dental, Lemgo, Germany	420	20,000 rpm
Diamond bur	Intensiv, Montagnola, Switzerland	15	200,000 rpm
Tungsten carbide bur (Q-Finisher)	Komet Dental, Lemgo, Germany	-	200,000 rpm
SofLex discs (aluminum oxide)	3 M ESPE, St Paul, MN, USA	coarse, medium, and fine, superfine	7400 rpm
SofLex + silicone polisher	3M ESPE, St Paul, MN, USA	coarse	7400 rpm
Coat (if recommended by manufacturer)	GC, Tokyo, Japan Voco, Cuxhaven, Germany	-	-

**Table 3:** Mean surface roughness (Sa) ± the respective standard deviations of the restorative materials by time points and finishing methods used.

		Surface Roughness (Sa) in μm		
Material	Finishing Method	Mean t1	Mean t2	Mean t3
CNF	No finishing	8.32 ± 2.52	9.49 ± 2.63	9.36 ± 3.01
	Tungsten carbide bur	8.58 ± 2.39	8.18 ± 1.87	8.39 ± 1.57
	Diamond bur	6.85 ± 1.55	6.40 ± 1.79	6.80 ± 1.63
	Arkansas bur	4.53 ± 1.92	4.95 ± 1.93	5.75 ± 2.36
	SofLex	2.05 ± 0.58	2.41 ± 0.74	2.69 ± 0.97
	SofLex + polishing	5.20 ± 2.30	5.44 ± 2.10	5.35 ± 2.21
DLF	No finishing	5.97 ± 1.75	6.29 ± 1.74	6.17 ± 1.69
	Tungsten carbide bur	2.12 ± 1.41	1.81 ± 0.71	1.63 ± 0.58
	Diamond bur	3.74 ± 1.45	3.99 ± 1.70	3.96 ± 1.65
	Arkansas bur	2.50 ± 1.12	2.18 ± 0.61	2.63 ± 1.16
	SofLex	5.59 ± 2.75	5.66 ± 2.07	6.18 ± 1.93
	SofLex + polishing	2.55 ± 0.71	2.71 ± 0.75	2.72 ± 0.73
EQF	No finishing	2.84 ± 2.09	3.24 ± 2.94	3.62 ± 3.07
	Tungsten carbide bur	5.98 ± 1.19	5.40 ± 0.74	6.69 ± 3.31
	Diamond bur	3.68 ± 1.33	3.92 ± 2.00	4.37 ± 2.23
	Arkansas bur	5.36 ± 1.84	5.03 ± 1.28	5.75 ± 1.43
	SofLex	4.10 ± 2.16	4.35 ± 3.29	4.33 ± 3.08
	SofLex + polishing	5.63 ± 2.12	5.73 ± 2.49	6.07 ± 2.92
	coat	2.26 ± 1.18	2.55 ± 1.34	5.66 ± 3.99
ISM	No finishing	4.18 ± 1.64	3.94 ± 2.11	3.98 ± 2.28
	Tungsten carbide bur	5.60 ± 1.99	6.54 ± 2.73	6.39 ± 2.28
	Diamond bur	6.40 ± 3.32	7.82 ± 4.39	7.53 ± 3.56
	Arkansas bur	4.23 ± 2.22	4.63 ± 2.07	4.80 ± 1.96
	SofLex	2.67 ± 0.58	2.70 ± 0.73	2.66 ± 0.72
	SofLex + polishing	3.14 ± 0.51	3.66 ± 1.25	3.63 ± 1.02
	coat	6.17 ± 2.53	6.32 ± 3.80	7.25 ± 4.68
KTU	No finishing	4.79 ± 2.94	5.71 ± 3.37	6.08 ± 3.06
	Tungsten carbide bur	6.37 ± 1.93	6.80 ± 2.40	6.43 ± 2.03
	Diamond bur	10.63 ± 2.47	11.22 ± 2.72	11.34 ± 3.03
	Arkansas bur	5.12 ± 2.50	4.96 ± 2.58	5.20 ± 2.77
	SofLex	1.72 ± 0.87	1.83 ± 0.78	1.92 ± 0.86
	SofLex + polishing	1.69 ± 0.51	1.66 ± 0.48	1.71 ± 0.50

CNF: Cention Forte, DLF: DeltaFil, EQF: Equia Forte HT, ISM: IonoStar Molar, KTU: Ketac Universal (KTU). t1: after finishing, t2: after 30 days, t3: after aging simulation.

**Table 4:** *p* values of the comparisons between finishing methods for the respective filling materials at time point 3.

		CNF	DLF	EQF	ISM	KTU
No finishing	Tungsten carbide bur	0.650	**<0.001**	**0.007**	**0.023**	1.000
	Diamond bur	**0.019**	**0.012**	0.174	**0.003**	**<0.001**
	Arkansas bur	0.059	**<0.001**	**0.013**	0.226	1.000
	SofLex	**<0.001**	1.000	0.226	0.174	**0.002**
	SofLex + polishing	**0.004**	**<0.001**	**0.013**	0.821	**0.001**
	Coat	-	-	0.112	0.082	-
Tungsten carbide bur	Diamond bur	0.059	**0.006**	0.059	0.597	**<0.001**
	Arkansas bur	**0.034**	1.000	0.545	0.096	1.000
	SofLex	**<0.001**	**<0.001**	**0.016**	**<0.001**	**<0.001**
	SofLex + polishing	**0.008**	1.000	0.326	**0.005**	**<0.001**
	Coat	-	-	0.151	0.705	-
Diamond bur	Arkansas bur	0.257	0.551	0.151	**0.019**	**<0.001**
	SofLex	**<0.001**	**0.011**	0.650	**<0.001**	**<0.001**
	SofLex + polishing	0.096	0.745	0.199	**<0.001**	**<0.001**
	Coat	-	-	0.705	0.597	-
Arkansas bur	SofLex	**0.001**	**<0.001**	**0.019**	**0.005**	**0.034**
	SofLex + polishing	0.545	1.000	0.940	0.199	**0.018**
	Coat	-	-	0.326	0.257	-
SofLex	SofLex + polishing	**0.002**	**<0.001**	**0.028**	**0.016**	1.000
	Coat	-	-	0.450	**0.013**	-
SofLex + polishing	Coat	-	-	0.326	0.112	-

Values in boldface indicate significant differences.

## Data Availability

The original contributions presented in this study are included in the article, and further inquiries can be directed to the corresponding author.

## Supplementary Materials

The following supporting information can be downloaded at: https://www.mdpi.com/article/10.3390/jfb15110325/s1.
