# Supplementary figures and images for: Effects of Finishing on Surface Roughness of Four Different Glass-Ionomer Cements and One Alkasite: In Vitro Investigation over Time Using Aging Simulation
Source: J Funct Biomater. 2024 Oct 31;15(11):325. doi: 10.3390/jfb15110325 (PMC11595664; doi:10.3390/jfb15110325)

| Material | T1                                                                                 | T2                                                                                  | T3                                                                                   |
|----------|------------------------------------------------------------------------------------|-------------------------------------------------------------------------------------|--------------------------------------------------------------------------------------|
| CNF      | 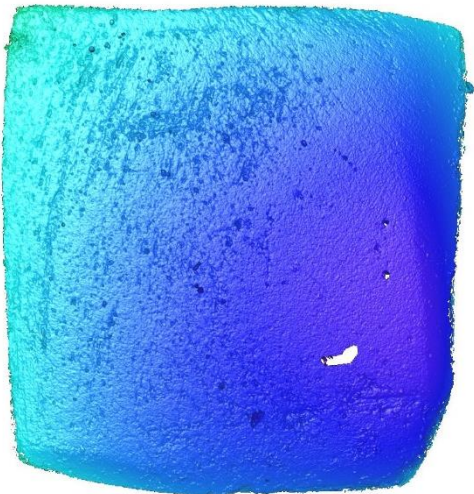  | 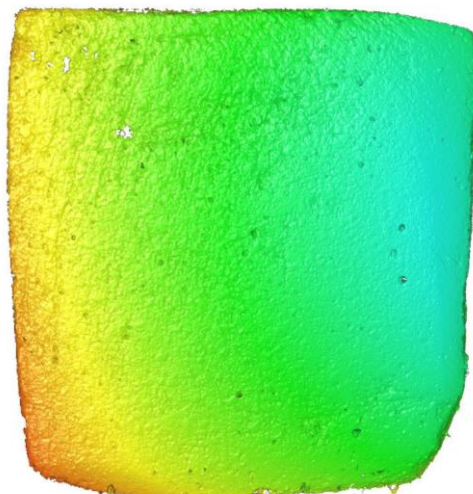  | 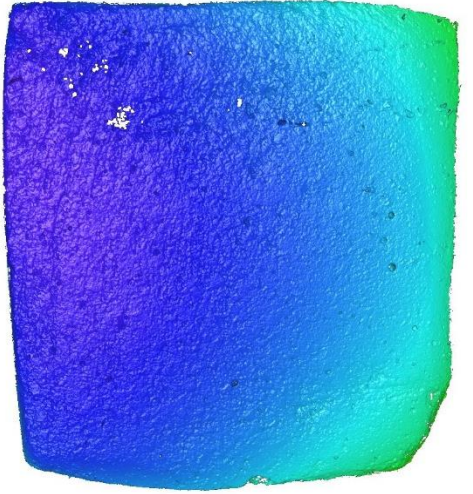  |
| DLF      | 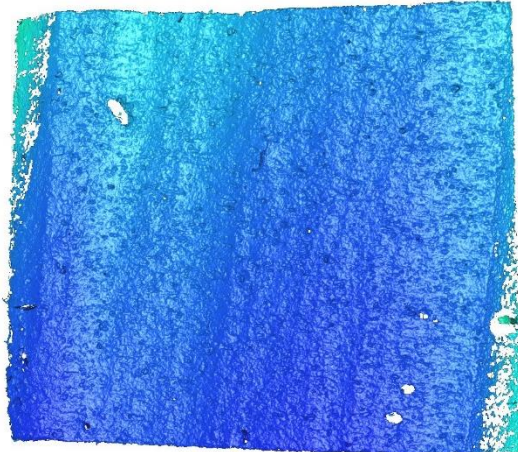 | 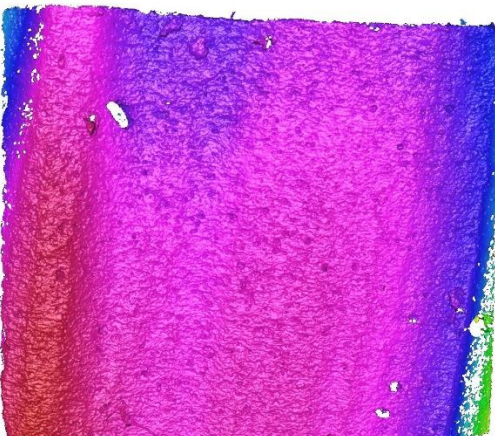 | 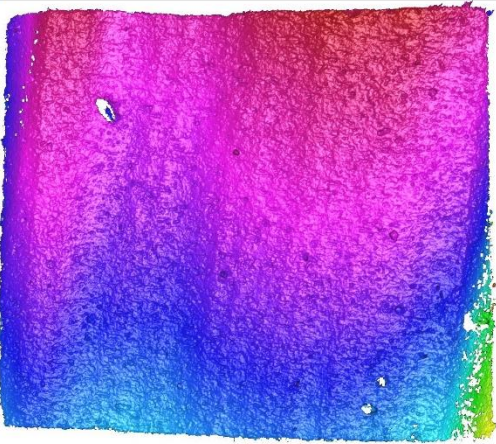 |

|     |                                                                                    |                                                                                     |                                                                                      |
|-----|------------------------------------------------------------------------------------|-------------------------------------------------------------------------------------|--------------------------------------------------------------------------------------|
| EQF | 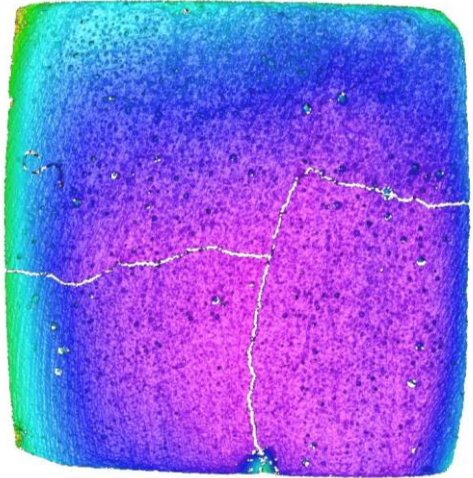  | 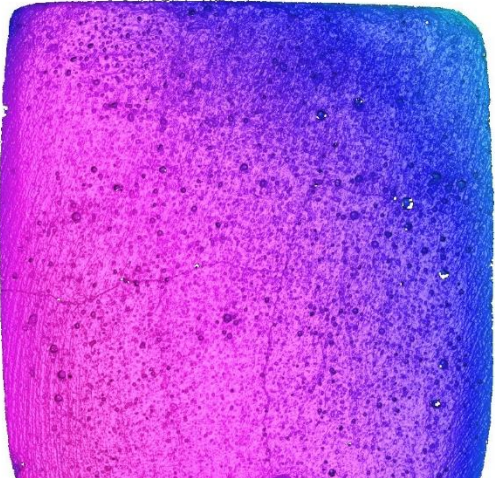  | 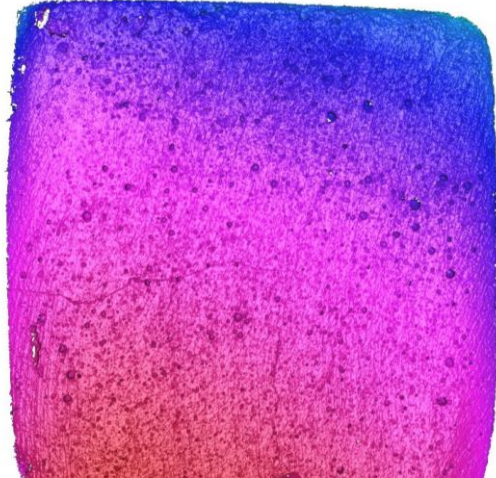  |
| ISM | 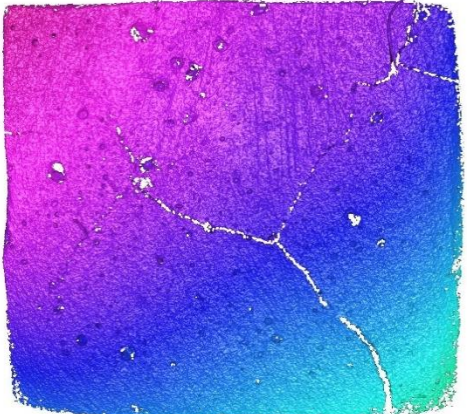 | 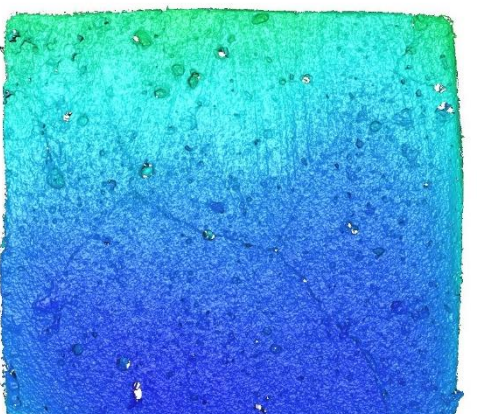 | 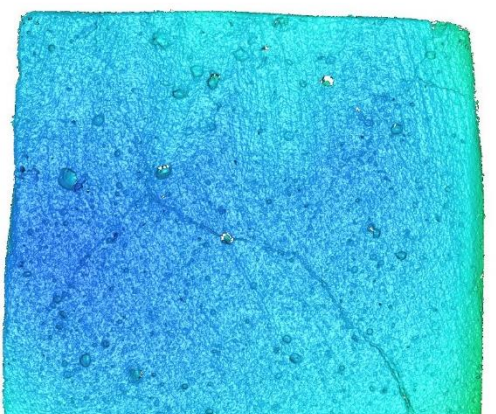 |

|     |                                                                                   |                                                                                    |                                                                                     |
|-----|-----------------------------------------------------------------------------------|------------------------------------------------------------------------------------|-------------------------------------------------------------------------------------|
| KTU | 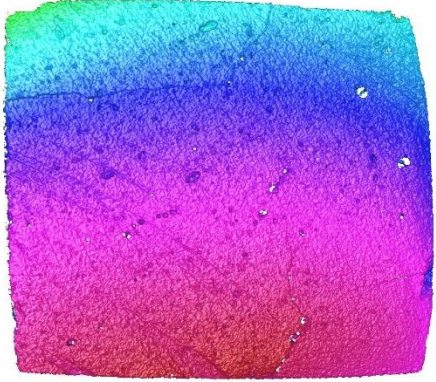 | 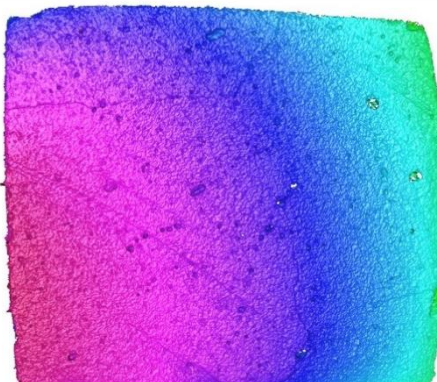 | 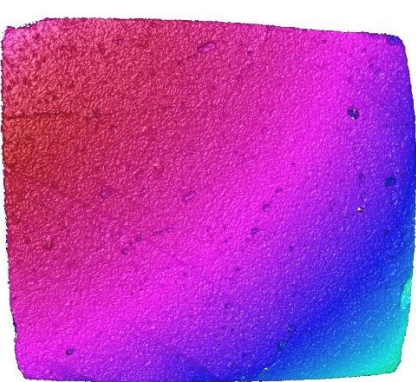 |
|-----|-----------------------------------------------------------------------------------|------------------------------------------------------------------------------------|-------------------------------------------------------------------------------------|

Supplement: Supplementary file 1 [file jfb-15-00325-s001.zip › jfb-3240807-supplementary.pdf]
